# Targeting Asparagine Metabolism in Well-Differentiated/Dedifferentiated Liposarcoma

**DOI:** 10.3390/cancers16173031

**Published:** 2024-08-30

**Authors:** Kyle D. Klingbeil, Blake R. Wilde, Danielle S. Graham, Serena Lofftus, Tyler McCaw, Nedas Matulionis, Sarah M. Dry, Joseph G. Crompton, Fritz C. Eilber, Thomas G. Graeber, David B. Shackelford, Heather R. Christofk, Brian E. Kadera

**Affiliations:** 1 Division of Surgical Oncology, Department of Surgery, University of California Los Angeles, 10833 Le Conte Ave, 54-117, Los Angeles, CA 90095, USA; 2 Jonsson Comprehensive Cancer Center, David Geffen School of Medicine, University of California Los Angeles, Los Angeles, CA 90095, USA; 3 Department of Biological Chemistry, University of California Los Angeles, Los Angeles, CA 90095, USA; 4 UCLA Metabolomics Center, University of California Los Angeles, Los Angeles, CA 90095, USA; 5 Department of Pathology, University of California Los Angeles, Los Angeles, CA 90095, USA; 6 Department of Molecular and Medical Pharmacology, David Geffen School of Medicine, University of California Los Angeles, Los Angeles, CA 90095, USA; 7 Department of Medicine, Division of Pulmonology and Critical Care, University of California Los Angeles, Los Angeles, CA 90095, USA

**Keywords:** well-differentiated/dedifferentiated liposarcoma, asparagine metabolism, mTORC1 signaling, ATF4, asparaginase, complex I inhibitor, electron transport chain, patient-derived organoids, patient-derived xenograft

## Abstract

**Simple Summary:**

Liposarcoma is a rare cancer of adipose tissue with limited treatment options. Here, we studied the fuel used by liposarcoma to develop a new strategy for treatment. Liposarcoma was found to rely on the amino acid Asparagine for tumor growth, especially in its most aggressive form. By combining treatments that limit both synthesis and uptake of Asparagine within liposarcoma cells, we demonstrated a unique sensitivity that reduced tumor growth in animal models. Altogether, findings from this study suggest that targeting Asparagine could be a promising new therapy in liposarcoma.

**Abstract:**

Background: mTORC1 activity is dependent on the presence of micronutrients, including Asparagine (Asn), to promote anabolic cell signaling in many cancers. We hypothesized that targeting Asn metabolism would inhibit tumor growth by reducing mTORC1 activity in well-differentiated (WD)/dedifferentiated (DD) liposarcoma (LPS). Methods: Human tumor metabolomic analysis was utilized to compare abundance of Asn in WD vs. DD LPS. Gene set enrichment analysis (GSEA) compared relative expression among metabolic pathways upregulated in DD vs. WD LPS. Proliferation assays were performed for LPS cell lines and organoid models by using the combination treatment of electron transport chain (ETC) inhibitors with Asn-free media. ^13^C-Glucose-labeling metabolomics evaluated the effects of combination treatment on nucleotide synthesis. Murine xenograft models were used to assess the effects of ETC inhibition combined with PEGylated L-Asparaginase (PEG-Asnase) on tumor growth and mTORC1 signaling. Results: Asn was enriched in DD LPS compared to WD LPS. GSEA indicated that mTORC1 signaling was upregulated in DD LPS. Within available LPS cell lines and organoid models, the combination of ETC inhibition with Asn-free media resulted in reduced cell proliferation. Combination treatment inhibited nucleotide synthesis and promoted cell cycle arrest. In vivo, the combination of ETC inhibition with PEG-Asnase restricted tumor growth. Conclusions: Asn enrichment and mTORC1 upregulation are important factors contributing to WD/DD LPS tumor progression. Effective targeting strategies require limiting access to extracellular Asn and inhibition of de novo synthesis mechanisms. The combination of PEG-Asnase with ETC inhibition is an effective therapy to restrict tumor growth in WD/DD LPS.

## 1. Introduction

Soft tissue sarcomas (STSs) are a heterogeneous group of connective tissue malignancies that arise from a mesenchymal precursor. They comprise about 1% of adult and 15% of pediatric malignancies with an estimated 13,000 new cases diagnosed in the United States each year [1]. While there are over fifty histological subtypes, liposarcoma (LPS) is among the most common in adults, accounting for 20% of cases [2]. LPS itself is subclassified into three major subtypes: (1) well-differentiated/dedifferentiated (WD/DD), (2) myxoid/round cell and (3) pleomorphic. WD/DD LPS is the most common subtype, characterized by the pathognomonic amplification of chromosome 12, including the cell cycle oncogenes *CDK4* and *MDM2*. The presence of DD within an LPS tumor portends a sixfold increase in mortality compared to tumors solely comprised of WD LPS [3]. Complete surgical resection remains the mainstay of treatment for WD/DD LPS, providing the greatest benefit in survival. Yet, surgery is limited to locoregional disease in patients with few comorbidities and acceptable functional status. External beam radiation, traditional chemotherapy and targeted small molecules have to date shown minimal improvement in survival for patients with locally advanced or metastatic WD/DD LPS [4]. Together, this paucity of effective therapy demonstrates the need for novel therapeutic strategies.

Metabolomics is the global assessment of small molecules, or metabolites, present within a biological system (e.g., cells, blood, or tissue). Comparison of the abundance of these metabolites under various physiologic conditions provides granularity to unique metabolic adaptations. As a known hallmark of cancer [5], recent efforts have developed novel strategies to target mechanisms of metabolic dysregulation using metabolomic techniques [6,7,8].

The dysregulation of Asparagine (Asn) metabolism has recently emerged as a critical player in tumor progression for many cancers [9,10,11,12]. Asn is a non-essential amino acid that has important roles in protein synthesis and cell signaling [11,12]. De novo production of Asn is catalyzed by asparagine synthetase (ASNS) through the unidirectional, ATP- and glutamine-dependent amidation of aspartate (Asp). Extracellular acquisition of Asn is less well understood, though it is predicted to be exchanged with other amino acids through the coordinated functions of multiple solute carriers [12]. Consistent with this idea, Asn plays a crucial role in regulating the uptake and cellular homeostasis of multiple amino acids [12].

Asn acts as a signaling molecule to regulate nutrient acquisition, cell proliferation, and tumor growth. ATF4 is an important transcription factor that increases the expression of ASNS and is activated by the depletion of intra-cellular Asn [11]. Asn also promotes mTORC1 activity, which regulates diverse cellular functions including synthesis of bioenergetic and biosynthetic substrates, enhanced proliferation, and promotion of pathological states such as cancer [11,12,13]. Together, Asn-dependent signaling has been found to be essential for cancer cell survival under conditions of limited nutrient availability [11]. Direct mTORC1 inhibition has displayed limited efficacy in many cancers due, in part, to compensatory resistance mechanisms previously described [14]. Targeting Asn metabolism may be more advantageous, as it affects multiple cellular processes and displays activity upstream of mTORC1 [11,12]. Whether Asn is essential for the proliferation and progression of WD/DD LPS remains unknown.

Mitochondrial respiration also contributes to the maintenance of amino acid homeostasis in cancer cells by generating biosynthetic precursors. While it was previously believed that proliferating cells require respiration primarily for the synthesis of Asp, which is rate-limiting in multiple biosynthetic pathways, recent evidence has suggested that respiration also contributes to the synthesis of Asp-derived Asn in order to promote mTORC1 signaling and maintenance of anabolism [11]. Cellular respiration can be impeded by targeting the electron transport chain (ETC). Most ETC inhibitors target mitochondrial Complex I, which represents the major entry point for electrons [15]. Blocking the ETC slows the TCA cycle and reduces the production of precursors used in the synthesis of Asn. In the context of ETC inhibition, cells become dependent on extracellular sources to maintain intra-cellular pools of Asn [11,12].

In this study, we sought to explore whether the dysregulation of Asn metabolism is important for tumor progression in WD/DD LPS. We found that WD/DD LPS tumors display higher Asn abundance relative to normal adipose, with an associated increase in mTORC1 activity. We targeted Asn pools by limiting both extracellular sources and de novo synthesis. This reduced in vitro proliferation of cell lines, growth of patient-derived organoids, and in vivo tumor growth. Altogether, this represents a rational targeted therapy regimen that demonstrates clinical potential.

## 2. Methods

### 2.1. GSEA Analysis

Our in-house gene expression microarray including both WD and DD LPS samples (n = 47) was retooled for this study [16]. In total, 288 metabolic pathway-related gene sets were selected and assessed using GSEA 4.1.0 software. Enrichment was analyzed for DD LPS relative to WD LPS samples. The gene sets were ranked by NES, and *p* < 0.05 and FDR (q) < 0.25 were used as thresholds for significance.

### 2.2. Cell Lines and Culture Conditions

LPS1, LPS2 and LPS3 cell lines were derived from dedifferentiated liposarcoma patient-derived xenografts and have also been previously validated [8]. 93T449 cell line (no. CRL-3043) was purchased from the ATCC (American Type Culture Collection, Manassas, VA, USA) and was derived from human WD LPS. Standard conditions for passaging were performed for all cell lines using DMEM with L-glutamine, 4.5 g/L glucose and sodium pyruvate (Corning, Corning, NY, USA; Cat#10013CV), supplemented with 10% (*v*/*v*) fetal bovine serum and 1% (*v*/*v*) penicillin–streptomycin (Thermo Fisher Scientific, Waltham, MA, USA; Cat#15140122). Cells were cultured in a 37 °C humidified, normoxic chamber with 5% CO_2_. All experiments were performed within the first 10 passages. Mycoplasma contamination was routinely assessed by PCR (Sigma-Aldrich, St. Louis, MO, USA; Cat#MP0035) or by direct confocal microscopy following incubation with the fluorescent dye, Hoescht 33342 (Thermo Fisher, Waltham, MA, USA; Cat#62249).

### 2.3. ETC Inhibition

Experiments involving ETC inhibition were performed using pyruvate-free DMEM with L-glutamine, 4.5 g/L of glucose (Thermo Fisher Scientific, Waltham, MA, USA; Cat#11965118), supplemented with 10% dialyzed fetal bovine serum (Thermo Fisher Scientific, Waltham, MA, USA; Cat#A3382001) and 1% penicillin–streptomycin. ETC inhibitors include metformin (Sigma-Aldrich, St. Louis, MO, USA; Cat#D150959), rotenone (Cayman Chemical, Ann Arbor, MI, USA; Cat#13995) and IACS-010597 (IACS) (Selleck Chem, Houston, TX, USA; Cat#S8731).

### 2.4. Cell Proliferation Analysis

Cells were seeded in triplicate or quadruplicate in white polystyrene 96-well plates (Corning, Corning, NY, USA; Cat#CLS3610-48EA) at 5 × 10^3^ cells per well and incubated under standard conditions overnight. Upon experiment initiation, cells were washed with dPBS (Corning, NY, USA; Cat#21030CVR) prior to medium replacement with pyruvate-free DMEM supplemented with or without 0.1 mM of asparagine in the presence of ETC inhibitor or equivalent volume of DMSO (vehicle). After 48 h of treatment, ATP concentration as a surrogate for relative cell count was quantified using a luminescence readout (Promega, Madison, WI, USA; Cat#G9242). Assessment of relative cell counts within the organoid model was completed similarly (Promega, Madison, WI, USA; Cat#G9681). Luminescence values were obtained using a Cytation 5 Imaging Reader (Agilent Technologies, Santa Claria, CA, USA). Raw values were normalized to the control mean to obtain relative values (0.1 mM of Asn and DMSO, unless otherwise stated).

### 2.5. MDM2 FISH Analysis

*MDM2* amplification was determined using in situ hybridization (ISH) protocols as recommended by Leica Biosystems. Sections were dewaxed, treated with BOND epitope retrieval solution 2 for 25 min (97 °C) followed by incubation with BOND enzyme (diluted to a final concentration of 1:3000) for 25 min (37 °C) using the Leica BOND Rx instrument (Leica Microsystems Inc., Buffalo Grove, IL, USA). The pretreatment protocol followed the manufacturer’s recommendations: Denaturation for 10 min, then ISH hybridization for 12 h. Kreatech FISH probes for MET (7q31 *MDM2* 12915 (Cat#12Q001V550) and SE12 (D12Z3) (Leica Biosystems, Amsterdam, Netherlands; Cat#12C002V495) were diluted into Bond hybridization Solution (Leica Biosystems, Amsterdam, Netherlands; Cat#AR9013). Anti-fade mounting medium with DAPI was added as a counterstain for DNA. Images were obtained using super-resolution confocal microscopy (LSM 880, Zeiss, Oberkochen, Germany) and processed using Airyscan methodology. Appropriate positive and negative controls were used, including morphological controls reviewed by expert pathologists. Samples were considered positive for *MDM2* amplification if they demonstrated diffuse nuclear staining or >50 dots per cell, as previously described [17].

### 2.6. Organoid Model

Patient-derived tumor organoids were established using the STAR protocol previously described [18]. Briefly, after selected patient’s underwent surgical resection, tumor samples were collected, rinsed in dPBS with antimycotic/antibiotic, and enzymatically digested using collagenase IV (ThermoFisher, Cat#17104019) for 1–3 h (37 °C). Erythrocytes were lysed using ammonium chloride (Stem Cell, Vancouver, Canada; Cat#07850). Cell suspensions were then filtered at 70 μm to remove debris and then counted. Mini-ring organoids were plated using 10 μL of cell suspension (5000 cells/well) in 1:1.33 pyruvate-free DMEM with 10% *v*/*v* dialyzed FBS to Matrigel. After incubation for 15 min (37 °C), organoids were submerged in selected medium conditions. Maxi-ring organoids were plated similarly using 70 μL of cell suspension at 150–200,000 cells/well. After 3–5 days of mini-ring organoid growth, media were aspirated, wells were washed with dPBS and drug treatments were given. Maxi-ring organoids were maintained in standard medium and imaged using automated confocal microscopy methods on a Cytation 5 Imaging Reader (Agilent Technologies, Santa Clara, CA, USA). Maxi-ring organoids were fixed in 10% neutral-buffered formalin for further analysis.

### 2.7. Glucose-Labeling and Metabolite Harvest

Cells were plated in six-well plates and metabolites extracted at the completion of the treatment timepoint, confluency 70–80%. For glucose-labeling experiments, pyruvate-free DMEM containing 10 mM U-^13^C-glucose (Cambridge Isotopes, Tewksbury, MA, USA; Cat#CLM-1396), L-glutamine and 10% dialyzed FBS in the presence of the specified treatment was used. Cells were washed twice with ice-cold wash buffer (150 mM of ammonium acetate, pH 7.3). Metabolites were extracted by incubation with 500 μL 80% MeOH at −80 °C. After 20 min, cells were scraped from the plates, transferred into Eppendorf tubes, vortexed and centrifuged at 17,000× *g*. A quantity of 250 μL of the resulting supernatant from each sample was dried in the presence of N_2_ using a nitrogen evaporator (N-EVAP, Organomation, Berlin, MA, USA), then stored at −80 °C until further analysis by liquid chromatography–mass spectrometry (LC-MS).

### 2.8. Cell Lysates and Western Blotting

Adherent cells were rinsed twice with dPBS, treated with preheated SDS Lysis Buffer (95 °C), scraped into an Eppendorf tube, heated for 5 min (95 °C), followed by sheering with a 27-gauged needle. Protein concentrations were quantified using a BCA Protein Assay Kit (ThermoFisher, Cat#23225) by following the manufacturer’s instructions. Sample analysis was completed using the spectrophotometer settings at 562 nm on a Cytation 5 Imaging Reader (Agilent Technologies, CA, USA).

Western blot analysis was performed using standard protocols as previously described [19], and the following primary antibodies were used: ASNS (Sigma-Aldrich, St Louis, MO, USA; Cat#HPA029318, 1:200), ATF4 (Abcam, Cambridge, United Kingdom; Cat#ab84909, 1:1000), pT389 S6 Kinase (Cell Signaling Technology, Danvers, MA, USA; Cat#9234, 1:500), S6 Kinase (Cell Signaling Technology, Danvers, MA, USA; Cat#2708, 1:1000), pS235/235 S6 ribosomal protein (Cell Signaling Technology, Danvers, MA, USA; Cat#4858, 1:3000), and S6 ribosomal protein (Cell Signaling Technology, Danvers, MA, USA; Cat#2217, 1:1000). Bound antibodies were visualized using ImobilonTM Western (Millipore Corporation, Billerica, MA, USA). Beta-actin (Cell Signaling Technology, Danvers, MA, USA; Cat#3700, 1:2000) and Vinculin (Abcam, Cambridge, United Kingdom; Cat#ab130007, 1:7500) served as loading controls, when appropriate. Western blot images were captured, and ImageJ software (NIH, Bethesda, Maryland, USA) was used for densitometric analysis. The intensity of each band was measured and normalized to the corresponding loading control. For each condition, the relative expression was calculated based on the percentage of the experimental control value, set at 100%. These values are reported under the band images.

### 2.9. Flow Cytometry Staining and Preparation

For cell cycle analysis experiments, after completion of treatment timepoint, cells were trypsinized, washed in cold dPBS and fixed in 70% ethanol for 24 h (−20 °C). Cells were then washed with 2% *v*/*v* FBS in dPBS twice and incubated in propidium iodide (Sigma-Aldrich, Cat#P4170) for 30 min in the dark. For apoptosis experiments, after completion of the treatment timepoint, cells were washed in dPBS, resuspended in 2% *v*/*v* FBS in dPBS and incubated in Annexin V-FITC (Thermo Fisher Scientific, Waltham, MA, USA; Cat#BDB556420) and propidium iodide (Sigma-Aldrich, St. Louis, MO, USA; Cat#P4170) working solution for 20 min in the dark. Annexin 1X binding buffer was then added. Appropriate positive and negative controls were used.

### 2.10. Flow Cytometry Acquisition and Analysis

Acquisition was completed using the AttuneNxT Cytometer (Thermo Fisher Scientific, Waltham, MA, USA). For cell cycle experiments, the PE channel EX561 and EM585/16 was used to detect PI. Linear scaling was used to detect PI signal. A total of 20,000–30,000 events were collected at medium to low speed (>200 μL/s). Analysis was completed using ModFit LT version 5.0.9 software. Pre-gates included FSC-A vs. SSC-A to remove debris and PI-A vs. PI-H to exclude aggregates. Manual analysis with the Diploid cell cycle model was used for determining G1, G2 and S Phase.

For apoptosis experiments, Annexin V-FITC conjugate was detected by the 488 nm laser with a 530/30 nm emission filter. PI was detected by the 561 nm laser using a 585/16 emission filter. No compensation was needed based on the single-color controls. Fluorescence intensities were measured on a logarithmic scale. The gating hierarchy included FSC-A vs. SSC-A parameters used to remove debris and FSC-H vs. FSC-A to remove aggregates.

### 2.11. Murine Tumor Model

NOD/SCID IL2γ null (NSG) mice were housed in pathogen-free facilities at UCLA, and care was provided by the Division of Laboratory Animal Medicine (DLAM) using biosafety level 2+ practices. Sample size calculations were used in order to determine the minimal number of subjects needed in each group, using power of 90% and alpha level of 0.05. Male and female mice (6–8 weeks of age) were used for drug trials, equally distributed among the treatment groups. Xenograft tumors were established by subcutaneous flank injection of 1 × 10^6^ LPS2 cells. Serial caliper measurements were obtained every other day, and tumor volumes were calculated using the formula, volume (mm^3^) = (length × width^2^)/2, where length defines the longest dimension and the width as the dimension perpendicular to length.

When xenograft tumors reached an average of 50–200 mm^3^, the mice were randomized and drug trials were initiated. Mouse weights were obtained weekly. Drug treatment continued until a mouse reached a humane endpoint or when a group mean tumor volume reached 1500 mm^3^. Mice were euthanized via CO_2_ inhalation followed by cervical dislocation. Endpoint mouse weight, tumor volume and tumor mass were measured. Sectioned xenograft tumors were snap-frozen in liquid nitrogen or fixed in 10% neutral-buffered formalin for 24 h.

### 2.12. Murine Model Drug Administration

For asparagine-adjusted diets, mice were given an asparagine-lacking diet (0% asparagine) or an asparagine-rich diet (4% asparagine), changed weekly. All diets were isonitrogenous and contained similar caloric densities. Asparaginase experiments utilized our institutionally synthesized PEGylated-Asparaginase (20 IU/kg in 0.9% NaCl) and 0.9% NaCl control delivered by intra-peritoneal injections weekly on Days 0, 7, 14 and 21. IACS-010759 (Selleck Chem, Houston, TX, USA; Cat#S8731) treatments were given MWF (10 mg/kg in 0.5% methylcellulose) and 0.5% methylcellulose control delivered by oral gavage. Metformin experiments were given ad libitum metformin in drinking water (250 mg/kg/day metformin with 0.5% *w*/*v* Splenda^®^, Heartland Food Products Group, Carmel, IN, USA) or 0.5% *w*/*v* Splenda^®^ control. Water was changed every 2–3 days.

### 2.13. Human Tumor Metabolite Extraction

Tumor fragments were cut on dry ice, weighed and added to pre-filled bead mill tubes (Thermo Fisher Scientific, Waltham, MA, USA; Cat#15340151). Metabolites were extracted in 1 mL 80% MeOH (−80 °C) using a Fisherbrand^TM^ bead mill homogenizer and centrifuged at 17,000× *g* (4 °C). Isolated supernatant volumes equivalent to 3 mg of tissue were then normalized to 500 μL using 80% MeOH and evaporated using a nitrogen evaporator (N-EVAP, Organomation Berlin, MA, USA; Cat#N45-35269). Samples were stored at −80 °C until metabolomic analysis.

### 2.14. Patient Tumor Metformin Abundance Analysis

Human tumor samples stored in our local tissue bank were selected based on whether patients were taking metformin at the time of surgical resection. Appropriate negative controls were also used. Tumor metabolites were extracted as previously described. A standard curve of known metformin concentrations was made in 80% MeOH supplemented with negative control tumor tissue and evaporated under nitrogen gas. Samples were stored at −80 °C until metabolomic analysis.

### 2.15. PEG-ASNase Synthesis

PEGylation of L-Asparaginase (ASNase) was initiated by incubating 650 units/mL *E. coli* ASNase (Sigma-Aldrich, St. Louis, MO, USA, Cat#A3809) and 175 mg/mL of N-hydroxylsuccinimide functionalized polyethylene glycol mPEG-NHS (Nanocs, Natick, MA, USA; Cat#PG1-SC-5k-1) in reaction buffer (0.9% NaCl, 10 mM of sodium phosphate, pH 7.4) overnight at 37 °C. PEGylated L-Asparaginase (PEG-ASNase) was isolated from unmodified ASNase by gel filtration using a HiLoad 16/600 Superdex 200 pg column (Sigma-Aldrich, St. Louis, MO, USA; Cat#GE28-9893-35) and a buffer composed of 0.9% NaCl. A combination of UV detection and SDS-PAGE was used to identify the fractions that contained PEG-ASNase, which were then pooled and filter-sterilized prior to injection into mice. Unmodified L-ASNase was also collected, pooled, and filter-sterilized to be used as a control for assessing the impact of PEGylation on catalytic activity.

PEG-ASNase activity was assessed using a colorimetric Asparaginase assay (Sigma). The ability of PEG-ASNase to clear plasma asparagine in mice was determined by tail vein injection of PEG-ASNase to female NSG mice (Jackson Laboratory, Bar Harbor, ME, USA) at 0, 20, 50, 150 and 300 units/kg. Blood was collected by retroorbital bleeding prior to injection and 1, 7 and 10 days following injection. Blood was transferred to EDTA-coated tubes, placed on ice and then centrifuged at 10,000× *g* (4 °C). The supernatant was extracted and then snap-frozen in liquid nitrogen, followed by storage at –80 °C until metabolomic analysis. The lowest effective dose of PEG-ASNase (20 units/kg) was further analyzed by IP injection. Blood was collected by retroorbital bleeding prior to injection and 7 days following injection. Serum was isolated from the blood and analyzed for asparagine and glutamine using LC-MS. For all remaining experiments, the lowest effective dose (20 units/kg) of PEG-ASNase was delivered via IP injection every 7 days.

### 2.16. Xenograft Tumor Immunohistochemistry and Analysis

Paraffin-embedded sections were cut at 4 μm thickness. Paraffin was removed with xylene and rehydrated through graded ethanol washes. Endogenous peroxidase activity was blocked with 3% *v*/*v* hydrogen peroxide in MeOH for 10 min. Heat-induced antigen retrieval (HIER) was carried out for all sections in AR9 buffer (Akoya Biosciences, Marlborough, MA, USA; Cat#AR9001KT) using a Biocare decloaker at 95 °C for 25 min. Appropriate morphological controls were used and reviewed by expert pathologists. The slides were then stained with anti-ATF4 (Abcam, Cambridge, United Kingdom; Cat#ab84909, 1:80), anti-ASNS (Sigma-Aldrich, St. Louis, MO, USA; Cat#HPA029318 1:200), anti-Phospho-4E-BP1 (Thr37/46) (Cell Signaling Technology, Danvers, MA, USA; Cat#2855, 1:1000), anit-p21 (Abcam, , Cambridge, United Kingdom; Cat#ab188224, 1:200) overnight (4 °C). Signal was detected using the DAKO EnVision^TM^ + DakoCytomation System Labelled Polymer HRP anti-rabbit (Agilent, Santa Clara, CA, USA; Cat#K4003). All sections were visualized with the diaminobenzidine reaction and counterstained with hematoxylin. Brightfield slides were digitally scanned on a ScanScope AT2 (Leica Biosystems, Vista, CA, USA) and analyzed using QuPath 0.2.3. Stain-specific algorithms were created to analyze positive and negative stained cells within each section. Thresholds were set to classify hematoxylin stain for nuclei and DAB stain for positive cellular staining.

### 2.17. Metabolomics

A mass spectrometry-based metabolomics approach was used as previously described [11]. Briefly, dried metabolites were reconstituted in 100 μL 50% (*v*/*v*) acetonitrile and dH_2_O solution. After centrifugation for 10 min at 17,000× *g*, 70 μL of supernatant was transferred to HPLC glass vials, and 10 μL of these metabolite solutions were injected per analysis. Metabolites were separated by liquid chromatography (Vanquish, Thermo Fisher Scientific, Waltham, MA, USA) and coupled to a mass spectrometer (Q-Exactive, Thermo Fisher Scientific, Waltham, MA, USA) to obtain ion chromatograms. Peaks were aligned among all samples and assigned identities using exact mass and retention time based on our in-house database. Peaks were quantified by area under the curve integration and normalized by the measured area, internal standard trifluoromethanesulfonate, and by cell count or tumor mass, as appropriate. Data were then exported as CSV files for further analysis. Experiments using stable isotope tracing underwent additional processing via the R package AccuCor to correct for natural isotope abundance.

### 2.18. Statistical Analysis

For statistical significance, a *p* value was set to <0.05 with 95% confidence interval. Continuous data with normal distribution in which the mean and standard deviation were known were compared by two-tailed *t* tests and ANOVA, as appropriate. Assessment of sample variance was completed by quantile–quantile plots. Comparisons between groups included in ANOVA were assessed by the Bonferroni Test. All statistical analyses were completed on GraphPad Prism 9.3.1 for MacOS. Data are presented as mean ± SEM, unless otherwise stated. Statistical significance is indicated as * *p* < 0.05, ** *p* < 0.01 and *** *p* < 0.001.

## 3. Results

**Figure 1:**
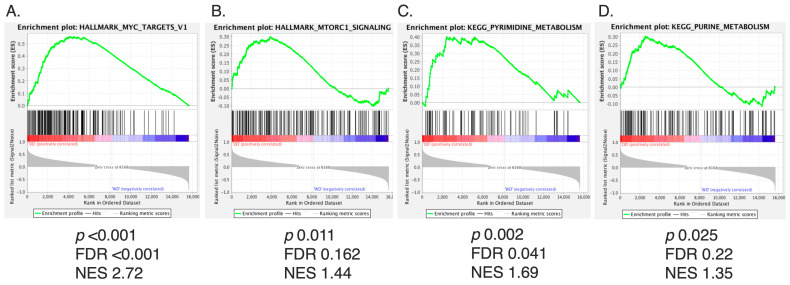
mTORC1 Signaling Among Top Gene Sets Enriched in DD LPS. Of the 288 metabolic pathway-related gene sets analyzed by GSEA, the top four ranked gene sets by NES are displayed. Gene sets were analyzed based on enrichment of DD LPS (n = 18) compared to WD LPS (n = 29). *p* < 0.05 and FDR (q) < 0.25 were used as cutoffs for significance. (**A**) Hallmark Myc Targets: NES 2.72, FDR < 0.001, *p* < 0.001, (**B**) Hallmark mTORC1 Signaling: NES 1.44, FDR 0.162, *p* = 0.011, (**C**) KEGG pyrimidine metabolism: NES 1.69, FDR 0.041, *p* = 0.002, and (**D**) KEGG purine metabolism: NES 1.35, FDR 0.22, *p* = 0.025.

We first aimed to explore whether mTORC1 activity was present in WD/DD LPS. Given that DD LPS is associated with a more rapid proliferation rate, we hypothesized that DD LPS would display higher activity of mTORC1 compared to WD LPS. To make this comparison, we utilized gene microarray data to evaluate the enrichment of gene sets in human DD LPS compared to WD LPS. The gene sets with the greatest enrichment in DD LPS were determined to be (1) Hallmark Myc Targets, (2) Hallmark mTORC1 signaling, (3) KEGG pyrimidine metabolism and (4) KEGG purine metabolism (Figure 1A–D, Supplementary Figure S1). Given that mTORC1 promotes Myc-dependent transcription and the expression of nucleotide biosynthesis genes, our GSEA data suggest that DD LPS displays increased mTORC1 signaling compared to WD LPS.

**Figure 2:**
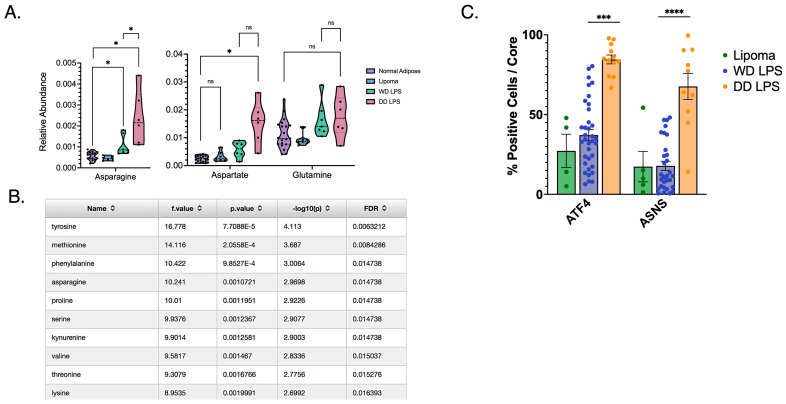
Increased Asparagine Abundance and Upregulated de novo Synthesis in DD LPS. (**A**) Human LPS samples freshly harvested from surgical resection and stored in liquid nitrogen underwent metabolomic analysis to compare relative abundance of key metabolites. Asn was found to be significantly more abundant among both WD LPS (*p* = 0.040) and DD LPS (*p* = 0.019) compared to adjacent normal adipose tissue. Additionally, Asn was significantly more abundant among DD LPS samples compared to WD LPS (*p* = 0.035). Each dot represents a unique patient sample. (**B**) Tabular format of metabolite comparisons between WD and DD LPS, ordered by *p*-value. Listed are the top 10 metabolites that significantly differed between WD and DD LPS. Notably, asparagine was ranked within the top four metabolites. (**C**) A tissue microarray was used to compare expression of de novo synthesis enzymes, ASNS and ATF4, among lipomatous tumors. IHC staining was quantified by cell positivity per tissue core. DD LPS was found to have significantly higher ATF4 cell positivity compared to WD LPS (*p* < 0.001) and ASNS cell positivity compared to WD LPS (*p* < 0.0001). Lipoma is a benign adipose tumor used as a control comparator. Each dot represents a unique patient sample. * *p* < 0.05, *** *p* < 0.001, **** *p* < 0.0001. ns = not significant.

mTORC1 and metabolism are mutually regulatory, and recent work has shown a role for Asn in signaling to mTORC1. We therefore evaluated WD and DD LPS tumors for differences in Asn abundance. Asn was found to have increased abundance in both WD and DD LPS compared to normal adipose tissue (Figure 2A). The benign comparator, lipoma, was similar to that of normal adipose tissue. Asp was also found to be uniquely elevated in DD LPS compared to normal adipose tissue (Figure 2A), whereas Gln was no different between groups. This importantly showed that DD LPS specifically increases the relative abundance of Asn, and its precursor Asp, suggesting the importance of de novo synthesis of Asn in DD LPS. The stepwise increase in abundance of Asn observed between WD and DD LPS also contributed to our conceptualization of the spectrum of this disease.

In order to perform an unbiased comparison of all metabolites identified by LC-MS, we ranked each metabolite and found that Asn was among the top four ranked metabolites significantly altered in DD LPS (Figure 2B). Asn is synthesized de novo by ASNS, whose expression is regulated by the activity of the nutrient-responsive transcription factor ATF4. We then evaluated the expression of ATF4 and ASNS in a human WD/DD LPS TMA to establish whether these tumors increase the abundance of Asn by increasing its synthetic mechanisms. Our evaluation of cell positivity established that while WD LPS displayed no difference in ATF4 or ASNS expression compared to lipoma, DD LPS highly expressed ATF4 and ASNS (Figure 2C). This suggests that the increased abundance of Asn observed in DD LPS may be the result of enhanced de novo synthesis mechanisms. As additional validation, we combined our genetic and metabolomic data using MetaboAnalyst in order to identify unique molecular pathways upregulated in DD LPS (Table 1). Among the top-ranked molecular pathways, the ‘alanine, aspartate, and glutamate metabolism’ pathway was highly upregulated in DD LPS.

**Figure 3:**
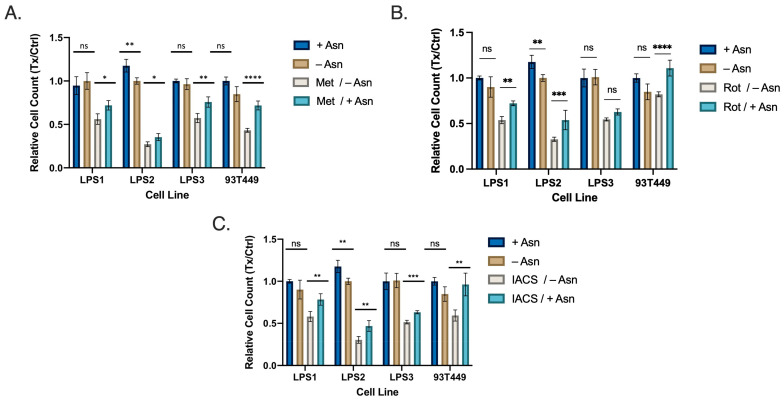
WD/DD LPS Display Sensitivity to Asparagine Depletion in vitro. Cell lines included DD LPS (LPS1, LPS2 and LPS3) and WD LPS (93T449). Cells were treated with media containing dialyzed FBS with supplemented 0.1 mM Asn (+Asn) or DMSO (−Asn). Cells were then treated with specified drug treatments for 48 h followed by quantification. (**A**) Metformin (2.5 mM) treatment combined with −Asn led to the greatest reduction in cell count compared to control. (**B**) Rotenone (25 nM), combined with −Asn treatment similarly resulted in the greatest reduction in cell count compared to control. (**C**) IACS (25 nM) demonstrated similar results. All +Asn vs −Asn/ETCi comparisons were significant with *p* < 0.001. * *p* < 0.05, ** *p* < 0.01, *** *p* < 0.001, **** *p* < 0.0001. ns = not significant.

Given that DD LPS displays (1) increased mTORC1 signaling, (2) increased abundance of Asn and (3) increased expression of ATF4 and ASNS, we next explored whether targeting Asn would restrict proliferation in WD/DD LPS. We inhibited the ETC in a panel of WD/DD LPS cell lines using several ETC inhibitors (ETCi) including metformin, rotenone and IACS-010759 (IACS). This was done with or without exogenous Asn supplementation at physiological concentrations. DD LPS cell lines LPS1, LPS2 and LPS3 were previously established at our center [8], and the WD LPS cell line 93T449 was obtained from ATCC. We found that when treated with the combination of ETCi in the absence of exogenous Asn, cell proliferation was significantly impacted in all cell lines investigated (Figure 3A–C). We also observed that in the presence of exogenous Asn, proliferation was partially restored in most instances. This adds to the developing body of literature which argues that the proliferative effects observed from ETCi can be partially explained by the depletion of Asn [11].

**Figure 4:**
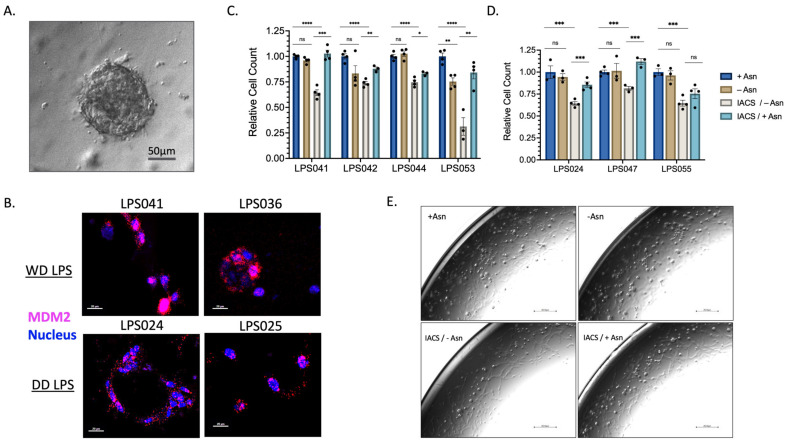
WD/DD LPS PDTOs Display Similar Sensitivity to Asparagine Depletion. PDTOs were generated following patient tumor harvest and grown in pyruvate-free DMEM with 10% *v*/*v* dialyzed FBS supplemented with 0.1 mM of Asn (+Asn) or DMSO (−Asn). Organoids were allowed to develop over 3–5 days prior to 72 h treatment with 50 nM IACS. (**A**) Representative PDTO viewed under light microscopy, scale bar 50 μm. (**B**) FISH analysis of PDTO’s using *MDM2* amplification as a marker of LPS cell validation. Magenta color denotes *MDM2* gene. Blue color denotes nuclear staining. Both WD and DD LPS PDTO samples demonstrate high amplification of *MDM2*, scale bar 20 μm. (**C**) WD LPS PDTOs, demonstrating combined 50 nM IACS/−Asn therapy, promotes the greatest reduction in organoid growth. LPS053 in particular was found to have a profound sensitivity to combined therapy. Each dot represents a technical replicate. (**D**) DD LPS PDTOs, demonstrating similar effects across all samples, in which combined IACS/−Asn therapy promotes the greatest reduction in organoid growth. Each dot represents a technical replicate. (**E**) LPS2 maxi-ring organoids treated with 72 h of 50 nM of IACS or DMSO (+/−Asn) were imaged under brightfield microscopy. Compared to +Asn/DMSO control, -Asn/IACS conditions demonstrated a lower total cell count and attenuated formation of organoid clusters, scale bar 251 μm. * *p* < 0.05, ** *p* < 0.01, *** *p* < 0.001, **** *p* < 0.0001. ns = not significant.

To investigate this effect further, we generated several patient-derived tumor organoids (PDTO) that better mimic the tumor microenvironment of WD/DD LPS (Figure 4A). PDTOs developed from both WD and DD LPS human samples were assessed for *MDM2* amplification to ensure that the outgrowth of tumor cells was maintained (Figure 4B). Again, we found that treatment with ETCi in the absence of exogenous Asn resulted in a significant reduction in PDTO growth (Figure 4C,D). In the presence of exogenous Asn, growth was partially restored. We noted variable responses across WD/DD LPS that were not specific to histologic subtype, adding to the known heterogeneity of this disease. Next, using brightfield microscopy, we assessed the three-dimensional architectural development of PDTOs following treatment with ETCi in the absence of exogenous Asn (Figure 4E). Qualitatively, we found that cells in the combination treatment group failed to form complex three-dimensional structures and remained spherical compared to control groups. Together, these data provide supporting evidence that WD/DD LPS displays sensitivity to Asn deprivation.

**Figure 5:**
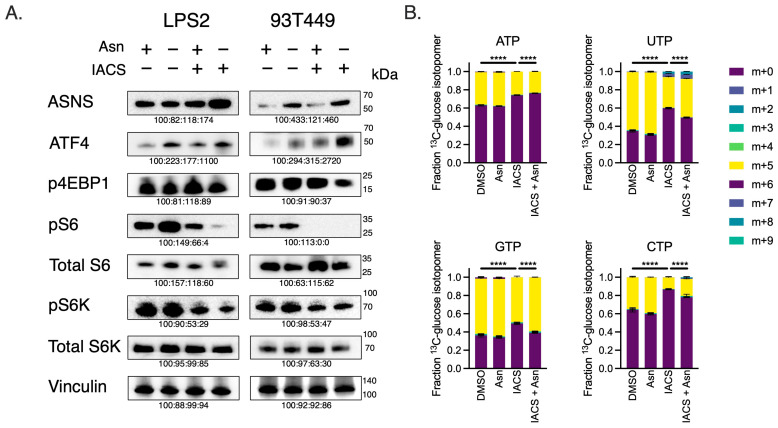
Asparagine Depletion Impairs mTORC1 Activity and Nucleotide Biosynthesis. (**A**) Western blots of LPS2 and 93T449 lysates 24 h post-treatment of 25 nM of IACS or DMSO in the absence or presence of 0.1 mM of Asn. Lysates were immunoblotted for ASNS, ATF4 and mTORC1 activation markers (pS6K, total S6K, pS6, total S6, p4EBP1). Vinculin was used as a loading control. (**B**) Fractional contributions of the various U-^13^C-glucose nucleotide isotopomers following 24 h treatment of 25 nM of IACS or DMSO in the absence or presence of 0.1 mM of Asn. The m + 0 isotopomer denotes lack of incorporation of the U-^13^C-glucose carbons into the specified nucleotide. The fraction of m+ > 0 denotes the rate of nucleotide biosynthesis. The combination of IACS and −Asn reduced the biosynthesis of all four nucleotides. **** *p* < 0.0001.

We next examined the mechanism of restricted proliferation observed in our pre-clinical models. Acknowledging that ETCi results in Asn depletion, we assessed ATF4 and mTORC1 activity in LPS2 and 93T449. Combination therapy with ETCi in the absence of exogenous Asn resulted in increased expression of ATF4 and ASNS (Figure 5A). Concurrently, in the presence of combination treatment, mTORC1 activity was diminished as assessed by the phosphorylation of downstream target proteins (pS6, pS6K, p4EBP1). When ETCi was administered in the presence of exogenous Asn, ATF4 and ASNS activity decreased, and mTORC1 activity was partially restored. These results demonstrate that the alterations in ATF4 and mTORC1 activity observed in WD/DD LPS under conditions of ETCi are due to Asn deprivation.

It has been previously shown that Asn promotes mTORC1-mediated nucleotide synthesis [12]. So, we assessed whether combination treatment of ETCi in the absence of exogenous Asn would inhibit nucleotide synthesis in WD/DD LPS, using glucose-labeling metabolomics (Figure 5B). Combination treatment indeed reduced the incorporation of radiolabeled carbons derived from universally labeled ^13^C-glucose into both purines and pyrimidines. This effect was partially restored in the presence of exogenous Asn for most nucleotides. These data suggest that combination treatment with ETCi in the absence of exogenous Asn results in concurrent inhibition of mTORC1 activity and nucleotide synthesis. Importantly, this also suggests that while Asp is often limiting for nucleotide synthesis, the effect of ETCi on nucleotide synthesis is due, at least in part, by the presence of Asn.

**Figure 6:**
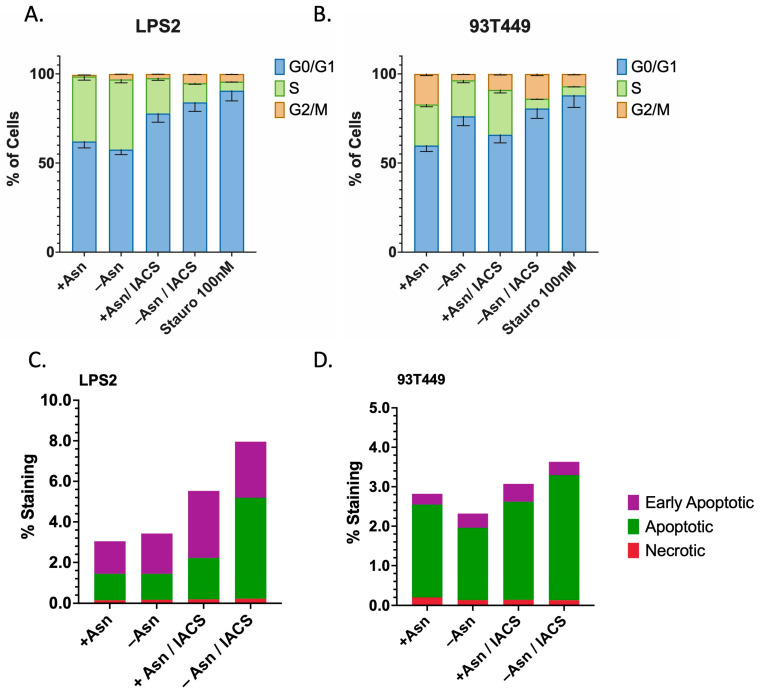
Asparagine Depletion Induces Cell Cycle Arrest and Apoptosis. (**A**) LPS2 and (**B**) 93T449 cells were treated with 25 nM of IACS or DMSO in the presence or absence of 0.1 mM of Asn for 24 h, followed by processing for FACS analysis to assess whether combination treatments inhibit cell growth by inducing cell cycle arrest (increasing fraction of cells in G_0_/G_1_ phase). A three-hour treatment with Staurosporine 100 nM was used as a positive control for cell cycle arrest. For both LPS2 and 93T449, combination treatment resulted in the highest fraction of cells in G_0_/G_1_ phase, indicating that cell cycle arrest does contribute to growth inhibition. This effect is partially reversed in the presence of Asn for both cell lines. (**C**) LPS2 and (**D**) 93T449 cells were similarly treated with 25 nM of IACS or DMSO in the presence or absence of 0.1 mM of Asn for 24 h, followed by processing for FACS analysis to assess whether combination treatments inhibit cell growth by inducing apoptosis. For both LPS2 and 93T449, combination treatment resulted in the highest rates of apoptosis, which was partially reversed in the presence of Asn.

Next, we sought to determine whether combination therapy limits proliferation through mechanisms of either apoptosis or cell cycle arrest. mTORC1 signaling is known to regulate both cell growth and cell cycle progression [20,21]. We therefore hypothesized that Asn targeting would preferentially result in cell cycle arrest. Indeed, using flow cytometry, we show that combination therapy results in the highest proportion of cells in G_0/1_ phase (Figure 6A, Supplementary Figure S2). ETCi in the presence of exogenous Asn results in the expansion of cells in S phase for 93T449, suggesting restoration of cell cycle progression. Interestingly, we found that in LPS2, ETCi in the presence of exogenous Asn only partially restored cell cycle progression. This suggests that LPS2 may display heightened sensitivity to ETCi as monotherapy due to mechanisms not currently understood.

Markers of apoptosis were also assessed under similar treatment conditions and demonstrated that combination therapy resulted in the highest levels of apoptotic and early apoptotic cells for both LPS2 and 93T449 (Figure 6B, Supplementary Figure S3). This effect appeared to be more strongly associated with LPS2, with a >2-fold increase in apoptosis with combination therapy. These data suggest cell cycle arrest as the primary mechanism for the perturbations in cell growth observed by treatment with ETCi in the absence of Asn. In addition, DD LPS may demonstrate enhanced sensitivity and may involve apoptotic pathways.

Bacterial orthologues of ASNase hydrolyze Asn and are used therapeutically to reduce circulating Asn and prevent its incorporation into proliferating cells. Acute lymphoblastic leukemia typically displays low-ASNS expression, such that the proliferation of lymphoblasts is dependent on the uptake of exogenous Asn, which is maintained at a concentration of 100 µM. Treatment with ASNase results in profound growth arrest and long-term remission [22]. As a result, the use of ASNase has become an integral treatment component in hematologic malignancies [23]. However, the use of ASNase in the treatment of solid malignancies has shown limited efficacy due to the high expression of ASNS [24]. Given that most DD LPS displays high ASNS expression (Figure 2C), we hypothesized that the combination of ASNase with ETCi may provide a pharmacologic means to effectively target Asn in WD/DD LPS.

**Figure 7:**
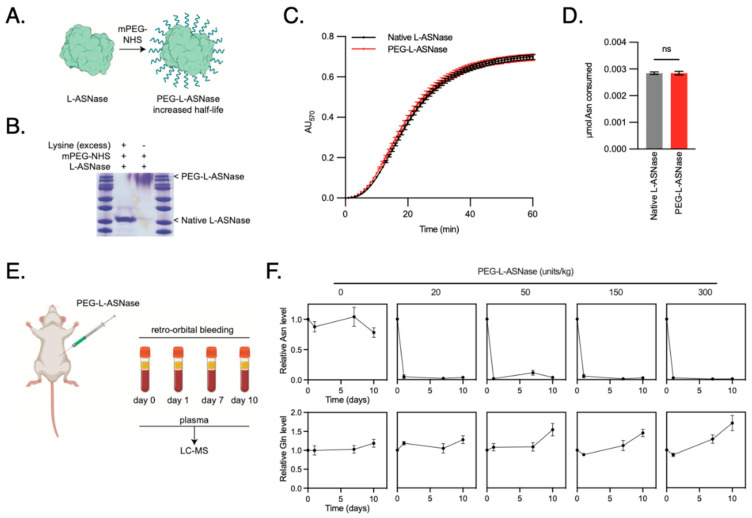
Synthesis and Validation of PEGylated Asparaginase for in vivo Drug Trials. (**A**) Schematic of the PEGylation design to improve stability in serum. (**B**) SDS-PAGE gel identifying the fractions of drug that contain PEG-ASNase following PEGylation. (**C**) Catalytic activity of PEG-ASNase was compared to the unmodified ASNase using a colorimetric Asparaginase assay and displayed similar activity rates. (**D**) Total amount of Asn consumed at the endpoint of the Asparaginase assay demonstrating no difference in activity after PEGylation. (**E**) Schematic of the experiment design, wherein PEG-ASNase was delivered to mice via tail vein injection at 0, 20, 50, 150 and 300 units/kg. Blood was collected by retroorbital bleeding prior to injection and 1, 7 and 10 days following injection. Plasma was isolated from the blood and analyzed for asparagine and glutamine using LC-MS. (**F**) Relative Asn levels from mice serum at the specified treatment dosing (mean ± SD). The lowest effective dose was 20 units/kg, which depleted Asn levels to at least 10 days. Treatment did not affect Gln levels, which is a known off-target effect of ASNase.

While ASNase was first identified in guinea pig serum, today the drug is primarily synthesized from bacterial sources [24]. Unfortunately, the stability of the drug in its original form is short-lived, requiring daily tail vein injections for murine models. Pharmaceutical companies have overcome this limitation by performing PEGylation to improve its stability. In effect, PEG-ASNase has allowed patients to receive intramuscular injections every 14–21 days rather than intravenous infusions three times a week [25], without therefore synthesized and tested PEG-ASNase for in vivo efficacy in reducing circulating Asn in our mouse models of LPS (Figure 7A). PEG-ASNase was isolated from unmodified ASNase by gel filtration (Figure 7B). PEG-ASNase maintained equivalent hydrolysis activity to unmodified ASNase (Figure 7C,D). Lastly, we assayed the ability of PEG-ASNase to clear plasma Asn in mice (Figure 7E). We found that the lowest effective dose for PEG-ASNase was 20 units/kg, and this resulted in a durable response through 10 days of observation with no measurable effects on Gln levels (Figure 7F). Given that off-target glutaminase activity is a known adverse effect of ASNase [26], we therefore proceeded with this dose of PEG-ASNase.

**Figure 8:**
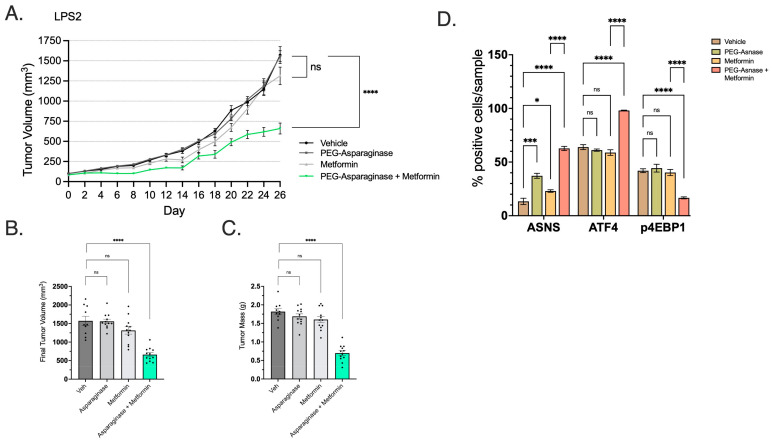
Combination of Metformin and PEG-ASNase Impairs Tumor Growth. (**A**) LPS2 tumor xenograft growth curves from treatment start date through endpoint. The combination of metformin with PEG-ASNase resulted in significant impairment of tumor growth compared to control (*p* < 0.0001). (**B**) Tumor volume at the study endpoint. Each dot represents an individual tumor xenograft. (**C**) Tumor mass at the study endpoint. Each dot represents an individual tumor xenograft. (**D**) IHC was performed on formalin-fixed tumor tissue and quantified by cell positivity/sample. Samples were probed for ASNS, ATF4 and the marker of mTORC1 activation (p4EBP1). ASNS and ATF4 positivity was significantly higher in the Met/PEG-ASNase group compared to controls. mTORC1 activity was also attenuated as shown by loss of p4EBP1 staining in the Met/PEG-ASNase group compared to controls. * *p* < 0.05, *** *p* < 0.001, **** *p* < 0.0001. ns = not significant.

We then explored the use of Asn deprivation as a therapeutic strategy in WD/DD LPS. Metformin is a known ETCi with acceptable oral bioavailability and prescribed frequently to patients with diabetes mellitus. We first evaluated the use of metformin with or without PEG-ASNase in an NSG murine model harboring LPS2 subcutaneous xenografts. The combination of PEG-ASNase and metformin significantly impaired tumor growth (Figure 8A–C). Neither treatment affected tumor growth independently. These results were comparable to other cancer models treated under similar conditions [11].

**Figure 9:**
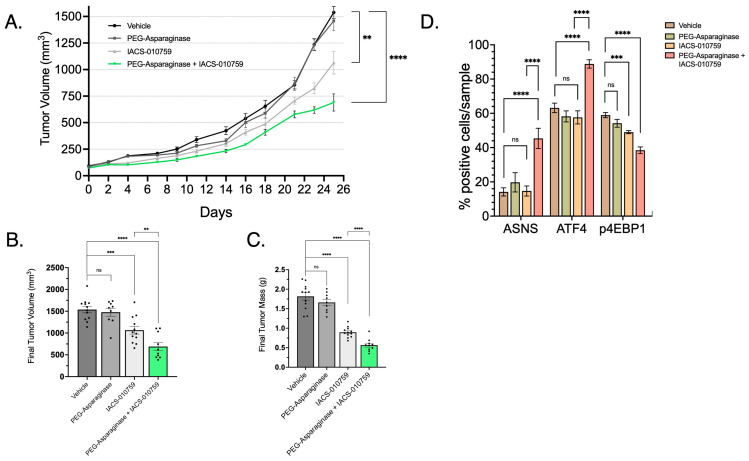
Combination of IACS and PEG-ASNase Impairs Tumor Growth. (**A**) LPS2 tumor xenograft growth curves from treatment start date through endpoint. The combination of IACS with PEG-ASNase resulted in significant impairment of tumor growth compared to control (*p* < 0.0001). IACS as monotherapy also resulted in a significant impairment of tumor growth (*p* < 0.01). (**B**) Tumor volume at the study endpoint. Each dot represents an individual tumor xenograft. (**C**) Tumor mass at the study endpoint. Each dot represents an individual tumor xenograft. (**D**) Quantified IHC of formalin-fixed tumor tissue probed for ASNS, ATF4 and a marker of mTORC1 activation (p4EBP1). ASNS and ATF4 expression was significantly higher in the IACS/PEG-ASNase group compared to controls. p4EBP1 expression was also attenuated in the Met/PEG-ASNase group compared to controls. IACS monotherapy resulted in a loss of p4EBP1, although did not affect ATF4 o ASNS expression. ** *p* < 0.01, *** *p* < 0.001, **** *p* < 0.0001. ns = not significant.

Using post mortem xenograft IHC, we assessed whether metformin/PEG-ASNase impacted ATF4 and mTORC1 activity. The combination of PEG-ASNase and metformin significantly increased expression of both ATF4 and ASNS while also showing a loss of mTORC1 activity (p4EBP1) (Figure 8D). Treatment with metformin in the absence of PEG-ASNase resulted in ATF4 and p4EBP1 expression levels similar to that of the vehicle group. We observed a similar growth impairment in LPS2 xenografts observed when PEG-ASNase was combined with the potent ETCi, IACS (Figure 9A–C). IACS is a preclinical drug, with oral bioavailability, which has previously been explored in early-phase clinical trials [27]. Asn signaling was similarly affected by combination treatment (Figure 9D). Interestingly, we also found that IACS treatment independently resulted in a significant reduction in tumor growth. This compares with our previously collected in vitro data, suggesting DD LPS displays enhanced sensitivity to ETCi.

**Figure 10:**
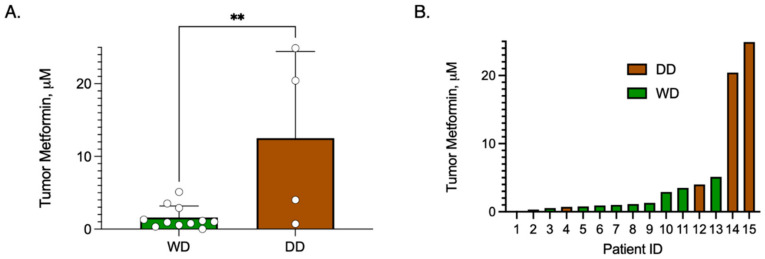
Metformin Concentration in Tumor Samples of Patients taking Metformin. Patients who were taking metformin at the time of surgical resection were included. Metformin concentrations were measured from cryobanked WD/DD LPS tissue samples (n = 15) using MS/LC. (**A**) Bar graph comparing mean ± SD metformin concentration by histologic group, WD vs. DD, noting that DD displayed significantly higher metformin compared to WD, *p* = 0.0074. Each dot represents a unique patient tumor sample. (**B**) Bar graph of tumor metformin concentration from each patient sample, noting high inter-subject variability. ** *p* < 0.01.

To determine whether achievable intra-tumoral metformin concentrations were achievable in WD/DD LPS, we performed metabolomics from cryobanked tumor tissue of patients taking metformin at the time of surgical resection (n = 15). By comparing intra-tumoral concentration by histologic subtype, we discovered that DD samples exhibited significantly higher metformin concentrations compared to WD samples, *p* = 0.0074 (Figure 10A). Inter-subject concentrations did vary widely, with a range of 0.01–24.9 μM (Figure 10B). Additional variables including metformin dosing, time from last dose, plasma concentration and normal tissue concentrations were not available for comparison. While these data are limited, they do provide preliminary evidence that metformin does accumulate in WD/DD LPS, with an observed preference for DD histology.

## 4. Discussion

By characterizing the metabolomic profiles of WD/DD LPS, we expanded our current knowledge by identifying key metabolites critical to tumor progression and unfettered proliferation. Among these key metabolites, Asn was found to be a fundamental purpose of mitochondrial respiration. At abundant levels, Asn signals to promote anabolic pathways. In its absence, biosynthesis is halted and cells retreat to senescence. Asn was also more abundant in DD LPS, which is characterized by enhanced proliferation rates, suggesting that this approach may provide benefit to patients.

WD/DD LPS cells obtain Asn through a variety of mechanisms. First, as a non-essential amino acid, Asn can be synthesized by cancer cells. The precursor for Asn, specifically Asp, is derived from mitochondrial respiration. By inhibiting respiratory pathways, Asp-derived Asn can be targeted. Second, cells exhibit the ability to uptake Asn from the microenvironment. Depletion of extracellular Asn can be accomplished through the administration of ASNase, which hydrolyzes Asn in the circulating bloodstream. By combining both targeting approaches, the signaling that promotes growth is lost, resulting in tumor growth restriction in WD/DD LPS.

WD/DD LPS represents a subset of STS with significant clinical challenges due to their resistance to conventional chemotherapies and potential for recurrence and metastasis. The current findings indicate that these tumors exhibit unique metabolic vulnerabilities that can be exploited for therapeutic benefit. Early-phase clinical trials are warranted to evaluate the safety, tolerability and efficacy of ETCi and Asnase combination therapy. These trials may provide critical insights into the therapeutic potential of this strategy and help identify biomarkers of response, ultimately providing more effective and personalized treatment approaches for patients with WD/DD LPS.

It is important to note that potent ETCis (e.g., IACS) have recently displayed limited clinical application due, in part, to unfavorable toxicity [27]. Conversely, metformin displays a wider therapeutic index, having been investigated in multiple clinical trials in solid tumors [28,29,30]. Unlike IACS, metformin weakly inhibits Complex I, thus reduces cellular energy production to a lesser extent. This partial inhibition may allow for a decrease in ATP production sufficient to stress cancer cells, while sparing normal cells that can better tolerate an energy deficit. Thus, metformin offers a potentially safer alternative in targeting mitochondrial metabolism and is therefore recommended for use in future clinical trial design until a newer generation of ETCis is developed.

Our evaluation of metformin concentration in patient tumor samples provided preliminary evidence that not only does metformin accumulate in WD/DD LPS but preferentially within the more aggressive DD subtype. This has clinical implications, further promoting the use of ETCi/Asnase combination therapy in advanced disease. Previous studies have observed similar findings of limited metformin uptake within adipose tissue compared to other tissues [31]. Metformin is a biguanide, and at physiologic pH in the serum exists as a hydrophilic cation, limiting its passive diffusion into peripheral tissues [31,32]. Uptake of metformin is enriched in cells that express cation transporters (SLC22A1/OCT1 and SLC22A3/OCT3) [33]. No prior studies have investigated the expression of these transporters in WD/DD LPS. The hydrophobicity of lipid-laden cells in WD LPS may serve as an alternative mechanism that reduces the uptake of metformin. Also, the observed inter-subject variability in the current study remains unresolved. Previous studies have associated metformin dosing and serum concentration with tumor concentration [31]. Future studies of WD/DD LPS should consider utilizing these comparisons in larger cohorts to confirm our initial findings.

## 5. Conclusions

Asn is an important player in maintaining amino acid homeostasis, that links functional respiration with mTORC1 signaling to enhance WD/DD LPS proliferation. Effective targeting strategies for WD/DD LPS require inhibition of both de novo synthesis and access to extracellular asparagine. The combination of Asparaginase with ETC inhibition is an effective therapy to inhibit tumor growth.

## Figures and Tables

**Table 1:** Multi-Omic Joint Pathway Analysis in WD/DD LPS.

Pathway Name	*p*-Value	FDR
Glycerophospholipid metabolism	1.77 × 10^−17^	2.12 × 10^−16^
Glycolysis or Gluconeogenesis	1.28 × 10^−11^	1.34 × 10^−10^
Phosphatidylinositol signaling system	6.55 × 10^−10^	6.12 × 10^−9^
Inositol phosphate metabolism	1.87 × 10^−9^	1.57 × 10^−8^
Glutathione metabolism	1.37 × 10^−8^	1.05 × 10^−7^
Retinol metabolism	3.62 × 10^−7^	2.54 × 10^−6^
Purine metabolism	6.01 × 10^−7^	3.88 × 10^−6^
Butanoate metabolism	6.10 × 10^−6^	3.66 × 10^−5^
Drug metabolism—other enzymes	1.92 × 10^−6^	1.06 × 10^−4^
Arginine biosynthesis	2.03 × 10^−5^	1.06 × 10^−4^
Pyruvate metabolism	2.46 × 10^−5^	1.22 × 10^−4^
Thiamine metabolism	5.48 × 10^−5^	2.56 × 10^−4^
Ascorbate and aldarate metabolism	1.10 × 10^−4^	4.90 × 10^−4^
alpha-Linolenic acid metabolism	1.94 × 10^−3^	8.16 × 10^−3^
Nicotinate and nicotinamide metabolism	3.36 × 10^−3^	1.28 × 10^−2^
Cysteine and methionine metabolism	3.37 × 10^−3^	1.28 × 10^−2^
Arginine and proline metabolism	6.70 × 10^−3^	2.45 × 10^−2^
Fructose and mannose metabolism	7.21 × 10^−3^	2.52 × 10^−2^
Citrate cycle (TCA cycle)	8.48 × 10^−3^	2.85 × 10^−2^
Histidine metabolism	8.94 × 10^−3^	2.89 × 10^−2^
Synthesis and degradation of ketone bodies	1.02 × 10^−2^	3.17 × 10^−2^
Alanine, aspartate and glutamate metabolism	1.69 × 10^−2^	5.06 × 10^−2^

Multi-Omic integration using transcriptomic and metabolomic data for human WD/DD LPS tumor samples was performed using the MetaboAnalyst software platform 5.0. Significant pathways are those that are enriched in DD LPS compared to WD LPS samples. *p*-value integration was performed using the Fisher’s method. Pathways are ordered by *p*-value, with *p*-value < 0.05 and FDR < 0.10 set as threshold values.

## Data Availability

The data presented in this study are available upon request from the corresponding author. The data are not publicly available due to patient privacy restrictions.

## Supplementary Materials

The following supporting information can be downloaded at: https://www.mdpi.com/article/10.3390/cancers16173031/s1, Figure S1: Heatmaps of Gene Sets included within GSEA Analysis; Figure S2: Flow Cytometry Peaks of Cell Cycle Analysis following Asparagine Depletion; Figure S3: Flow Cytometry Gating for Apoptosis following Asparagine Depletion.

## Footnotes

This work was presented virtually at the Connective Tissue Oncology Society (CTOS) 2021 Annual Meeting.

## References

[1] Siegel R.L., Miller K.D., Fuchs H.E., Jemal A. (2022). Cancer statistics, 2022. CA Cancer J. Clin..

[2] Dei Tos A.P. (2000). Liposarcoma: New entities and evolving concepts. Ann. Diagn. Pathol..

[3] Qin C., Yang G., Yang J., Ren B., Wang H., Chen G., Zhao F., You L., Wang W., Zhao Y. (2020). Metabolism of pancreatic cancer: Paving the way to better anticancer strategies. Mol. Cancer.

[4] Eilber F.C., Brennan M.F., Riedel E., Alektiar K.M., Antonescu C.R., Singer S. (2005). Prognostic factors for survival in patients with locally recurrent extremity soft tissue sarcomas. Ann. Surg. Oncol..

[5] Schmidt D.R., Patel R., Kirsch D.G., Lewis C.A., Vander Heiden M.G., Locasale J.W. (2021). Metabolomics in cancer research and emerging applications in clinical oncology. CA Cancer J. Clin..

[6] Vernieri C., Casola S., Foiani M., Pietrantonio F., de Braud F., Longo V. (2016). Targeting cancer metabolism: Dietary and pharmacologic interventions. Cancer Discov..

[7] Luengo A., Gui D.Y., Vander Heiden M.G. (2017). Targeting metabolism for cancer therapy. Cell Chem. Biol..

[8] Braas D., Ahler E., Tam B., Nathanson D., Riedinger M., Benz M.R., Smith K.B., Eilber F.C., Witte O.N., Tap W.D. (2012). Metabolomics strategy reveals subpopulation of liposarcomas sensitive to gemcitabine treatment. Cancer Discov..

[9] Hettmer S., Schinzel A.C., Tchessalova D., Schneider M., Parker C.L., Bronson R.T., Richards N.G., Hahn W.C., Wagers A.J. (2015). Functional genomic screening reveals asparagine dependence as a metabolic vulnerability in sarcoma. Elife.

[10] Knott S.R.V., Wagenblast E., Khan S., Kim S.Y., Soto M., Wagner M., Turgeon M.O., Fish L., Erard N., Gable A.L. (2018). Asparagine bioavailability governs metastasis in a model of breast cancer. Nature.

[11] Krall A.S., Mullen P.J., Surjono F., Momcilovic M., Schmid E.W., Halbrook C.J., Thambundit A., Mittelman S.D., Lyssiotis C.A., Shackelford D.B. (2021). Asparagine couples mitochondrial respiration to ATF4 activity and tumor growth. Cell Metab..

[12] Krall A.S., Xu S., Graeber T.G., Braas D., Christofk H.R. (2016). Asparagine promotes cancer cell proliferation through use as an amino acid exchange factor. Nat. Commun..

[13] Leung-Pineda V., Kilberg M.S. (2002). Role of Sp1 and Sp3 in the nutrient-regulated expression of the human asparagine synthetase gene. J. Biol. Chem..

[14] Laplante M., Sabatini D.M. (2012). mTOR signaling in growth control and disease. Cell.

[15] Sharma L.K., Lu J., Bai Y. (2009). Mitochondrial respiratory complex I: Structure, function and implication in human diseases. Curr. Med. Chem..

[16] Tap W.D., Eilber F.C., Ginther C., Dry S.M., Reese N., Barzan-Smith K., Chen H.W., Wu H., Eilber F.R., Slamon D.J. (2011). Evaluation of well-differentiated/de-differentiated liposarcomas by high-resolution oligonucleotide array-based comparative genomic hybridization. Genes Chromosomes Cancer.

[17] Kulkarni A.S., Wojcik J.B., Chougule A., Arora K., Chittampalli Y., Kurzawa P., Mullen J.T., Chebib I., Nielsen G.P., Rivera M.N. (2019). MDM2 RNA In Situ Hybridization for the Diagnosis of Atypical Lipomatous Tumor: A Study Evaluating DNA, RNA, and Protein Expression. Am. J. Surg. Pathol..

[18] Eilber F.C., Eilber F.R., Eckardt J., Rosen G., Riedel E., Maki R.G., Brennan M.F., Singer S. (2004). The impact of chemotherapy on the survival of patients with high-grade primary extremity liposarcoma. Ann. Surg..

[19] Klingbeil K.D., Tang J.P., Graham D.S., Lofftus S.Y., Jaiswal A.K., Lin T.L., Frias C., Chen L.Y., Nakasaki M., Dry S.M. (2023). IGF2BP3 as a Prognostic Biomarker in Well-Differentiated/Dedifferentiated Liposarcoma. Cancers.

[20] Jacinto E., Hall M.N. (2003). Tor signalling in bugs, brain and brawn. Nat. Rev. Mol. Cell Biol..

[21] Fingar D.C., Richardson C.J., Tee A.R., Cheatham L., Tsou C., Blenis J. (2004). mTOR controls cell cycle progression through its cell growth effectors S6K1 and 4E-BP1/eukaryotic translation initiation factor 4E. Mol. Cell. Biol..

[22] Avramis V.I., Tiwari P.N. (2006). Asparaginase (native ASNase or pegylated ASNase) in the treatment of acute lymphoblastic leukemia. Int. J. Nanomed..

[23] Egler R.A., Ahuja S.P., Matloub Y. (2016). L-asparaginase in the treatment of patients with acute lymphoblastic leukemia. J. Pharmacol. Pharmacother..

[24] Van Trimpont M., Peeters E., De Visser Y., Schalk A.M., Mondelaers V., De Moerloose B., Lavie A., Lammens T., Goossens S., Van Vlierberghe P. (2022). Novel Insights on the Use of L-Asparaginase as an Efficient and Safe Anti-Cancer Therapy. Cancers.

[25] Fu C.H., Sakamoto K.M. (2007). PEG-asparaginase. Expert Opin. Pharmacother..

[26] Chan W.K., Lorenzi P.L., Anishkin A., Purwaha P., Rogers D.M., Sukharev S., Rempe S.B., Weinstein J.N. (2014). The glutaminase activity of L-asparaginase is not required for anticancer activity against ASNS-negative cells. Blood J. Am. Soc. Hematol..

[27] Yap T.A., Daver N., Mahendra M., Zhang J., Kamiya-Matsuoka C., Meric-Bernstam F., Kantarjian H.M., Ravandi F., Collins M.E., Francesco M.E.D. (2023). Complex I inhibitor of oxidative phosphorylation in advanced solid tumors and acute myeloid leukemia: Phase I trials. Nat. Med..

[28] Bilusic M., Toney N.J., Donahue R.N., Wroblewski S., Zibelman M., Ghatalia P., Ross E.A., Karzai F., Madan R.A., Dahut W.L. (2022). A randomized phase 2 study of bicalutamide with or without metformin for biochemical recurrence in overweight or obese prostate cancer patients (BIMET-1). Prostate Cancer Prostatic Dis..

[29] Martinez J.A., Chalasani P., Thomson C.A., Roe D., Altbach M., Galons J.P., Stopeck A., Thompson P.A., Villa-Guillen D.E., Chow H.H. (2016). Phase II study of metformin for reduction of obesity-associated breast cancer risk: A randomized controlled trial protocol. BMC Cancer.

[30] Skinner H., Hu C., Tsakiridis T., Santana-Davila R., Lu B., Erasmus J.J., Doemer A.J., Videtic G.M.M., Coster J., Yang A.X. (2021). Addition of Metformin to Concurrent Chemoradiation in Patients With Locally Advanced Non-Small Cell Lung Cancer: The NRG-LU001 Phase 2 Randomized Clinical Trial. JAMA Oncol..

[31] Phillips J.D., Pooler D.B., Ness D.B., Fay K., Tau S., Demidenko E., Hampsch R.A., Lewis L.D., Miller T.W. (2023). Tumour, whole-blood, plasma and tissue concentrations of metformin in lung cancer patients. Br. J. Clin. Pharmacol..

[32] Graham G.G., Punt J., Arora M., Day R.O., Doogue M.P., Duong J.K., Furlong T.J., Greenfield J.R., Greenup L.C., Kirkpatrick C.M. (2011). Clinical pharmacokinetics of metformin. Clin. Pharmacokinet..

[33] Shu Y., Sheardown S.A., Brown C., Owen R.P., Zhang S., Castro R.A., Ianculescu A.G., Yue L., Lo J.C., Burchard E.G. (2007). Effect of genetic variation in the organic cation transporter 1 (OCT1) on metformin action. J. Clin. Investig..

