# Supplementary material for: Targeting Asparagine Metabolism in Well-Differentiated/Dedifferentiated Liposarcoma
Source: Cancers (Basel). 2024 Aug 30;16(17):3031. doi: 10.3390/cancers16173031 (PMC11394161; doi:10.3390/cancers16173031)

Supplementary

Figure S1. Heatmaps of Gene Sets included within GSEA Analysis.

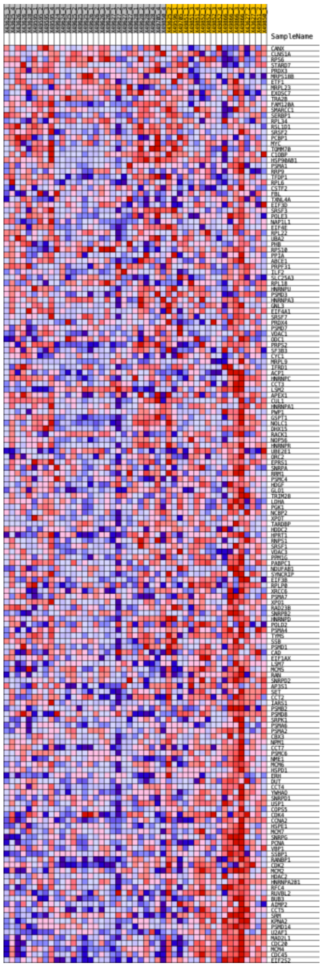

A: HALLMARK MYC TARGET V1

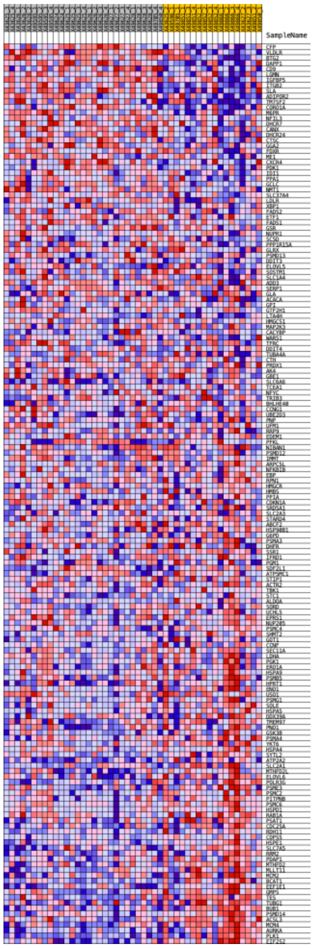

B: HALLMARK MTORC1 SIGNALING.

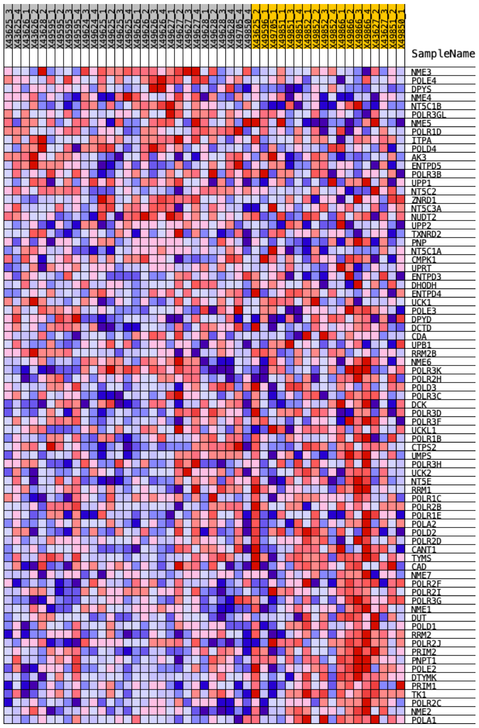

C: KEGG PYRIMIDINE METABOLISM.

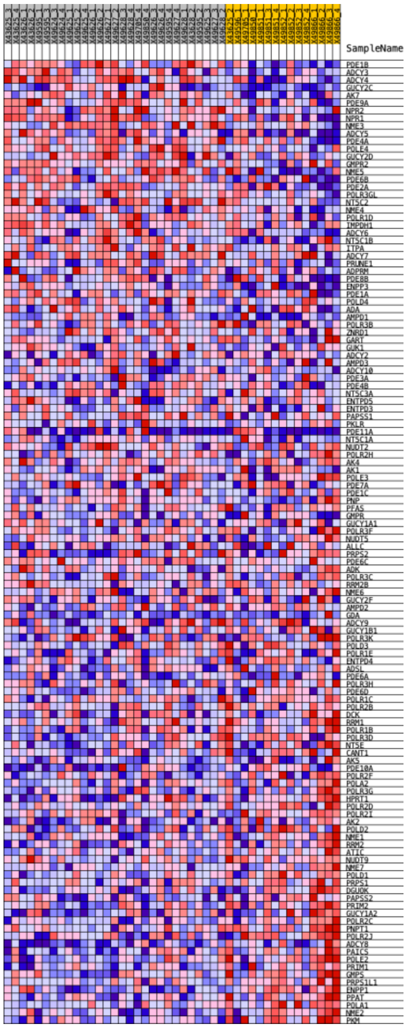

D: KEGG PURINE METABOLISM.

Figure S2. Flow Cytometry Peaks of Cell Cycle Analysis following Asparagine Depletion.

A. 93T449 - +Asn / DMSO

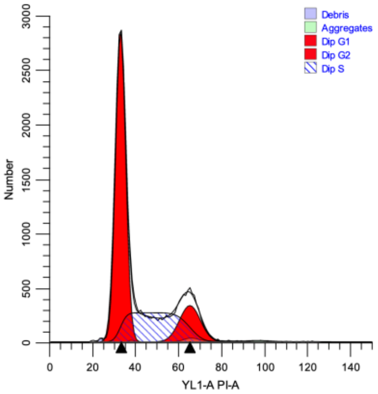

C. 93T449- +Asn / IACS

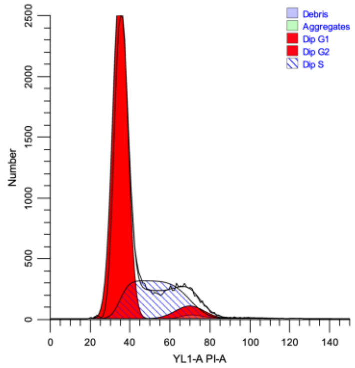

B. 93T449 - -Asn / DMSO

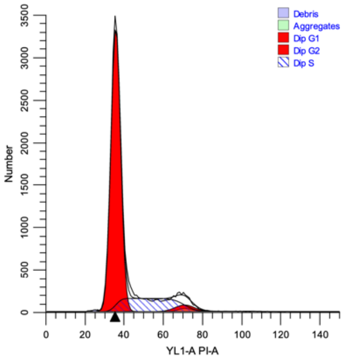

D. 93T449- -Asn / IACS

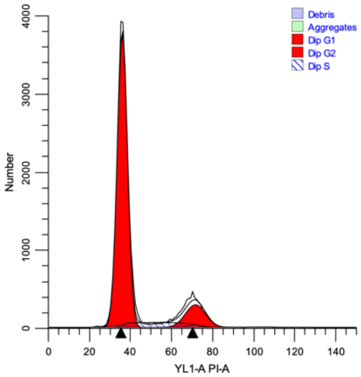

E. 93T449 - Staurosporine

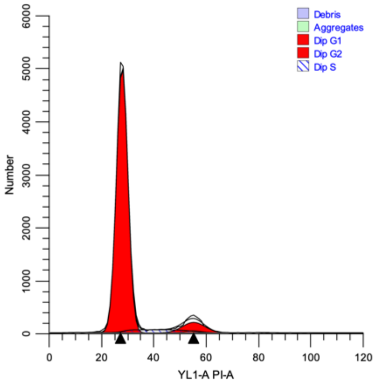

F. LPS2 - +Asn / DMSO

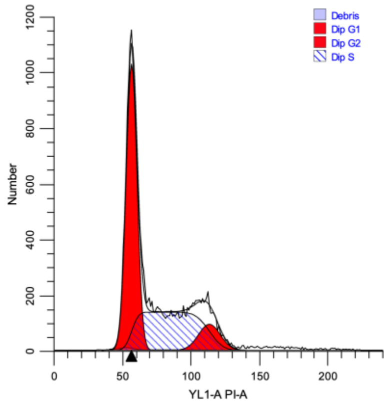

G. LPS2 - -Asn / DMSO

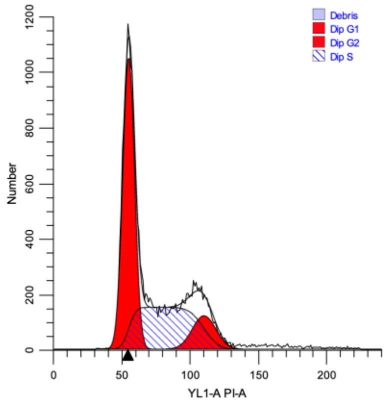

H. LPS2 - +Asn / IACS

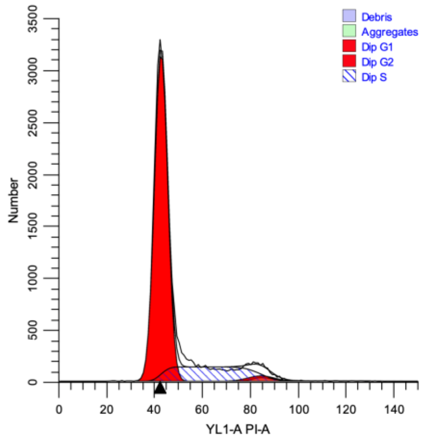

I. LPS2 - -Asn / IACS

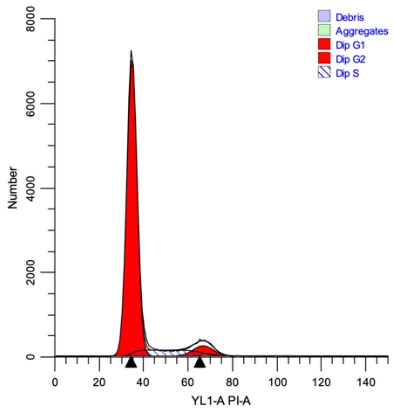

J. LPS2 - Staurosporine

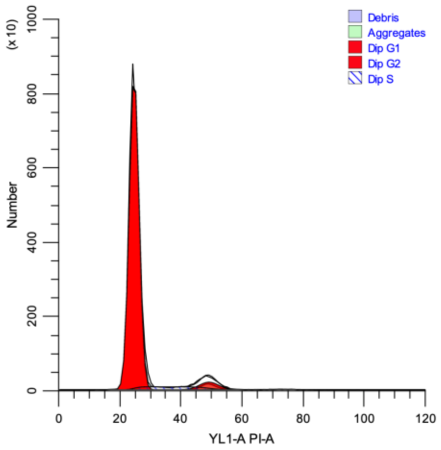

Figure S3. Flow Cytometry Gating for Apoptosis following Asparagine Depletion.

A. 93T449 - +Asn / DMSO

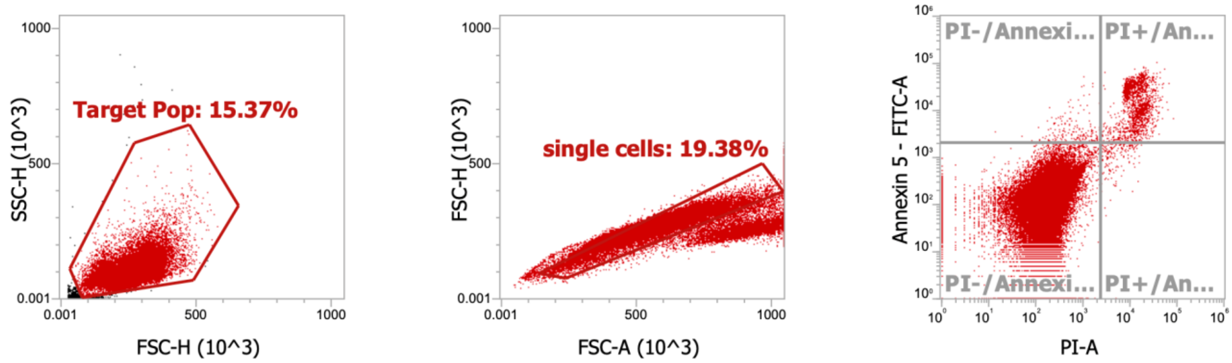

B. 93T449 - -Asn / DMSO

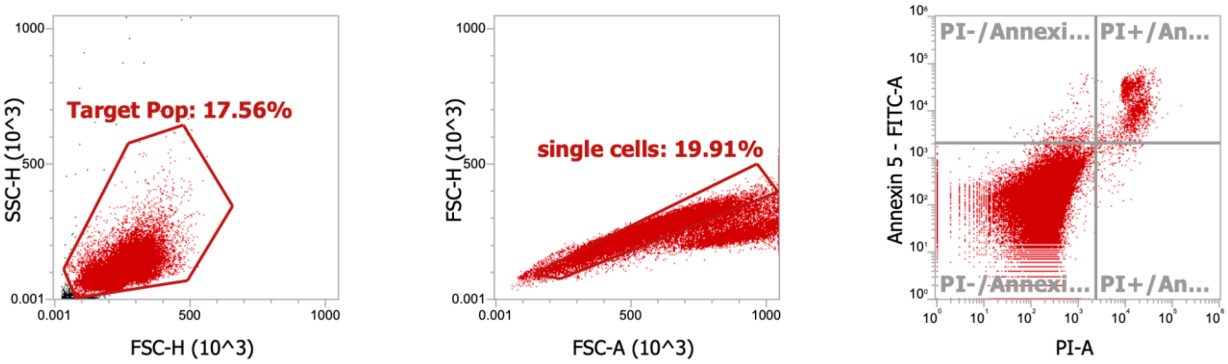

C. 93T449 - +Asn / IACS

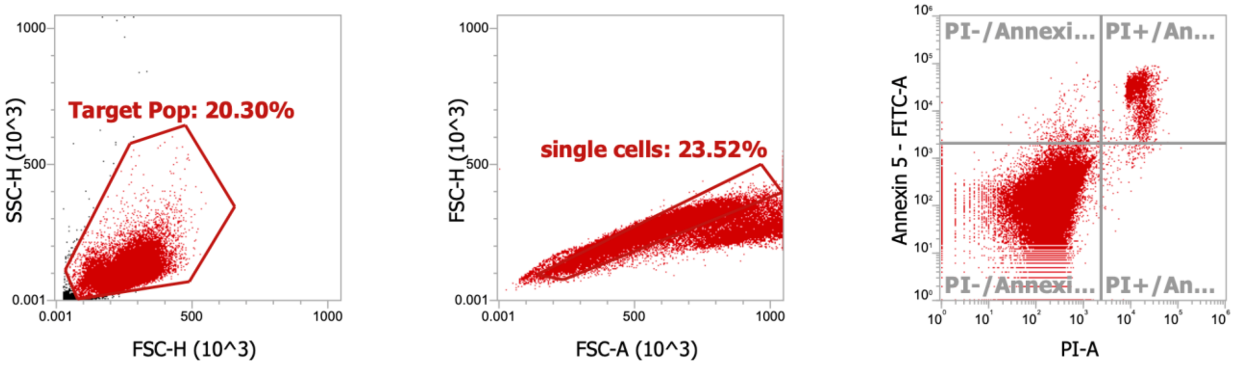

D. 93T449 - -Asn / IACS

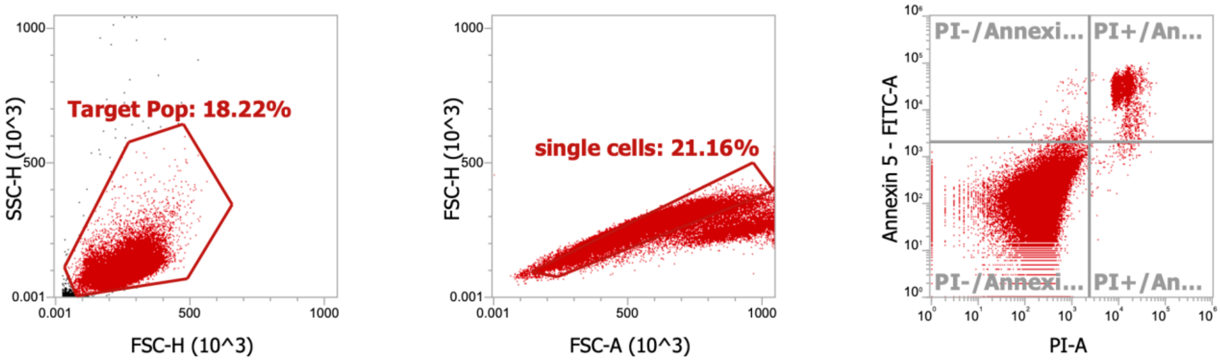

E. LPS2 - +Asn / DMSO

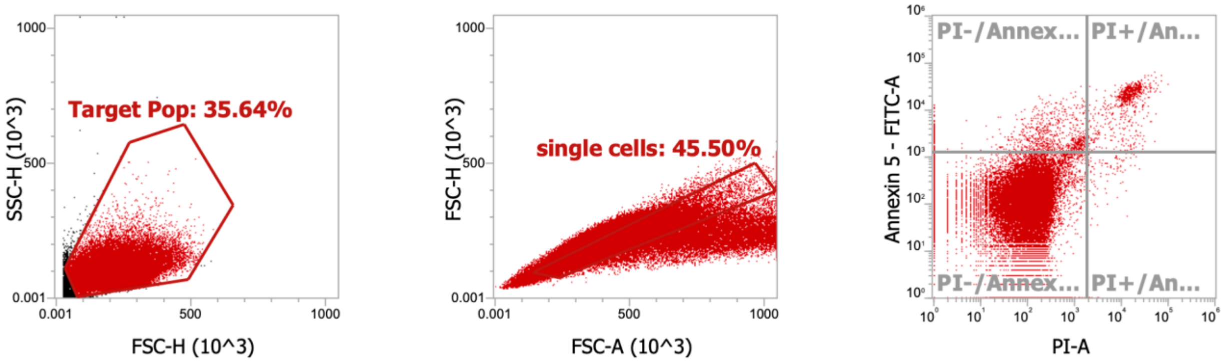

F. LPS2 - -Asn / DMSO

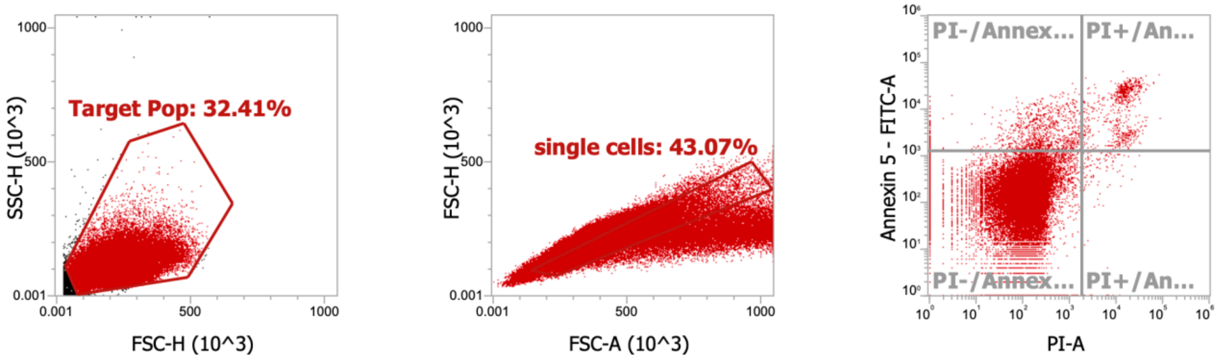

G. LPS2 - +Asn / IACS

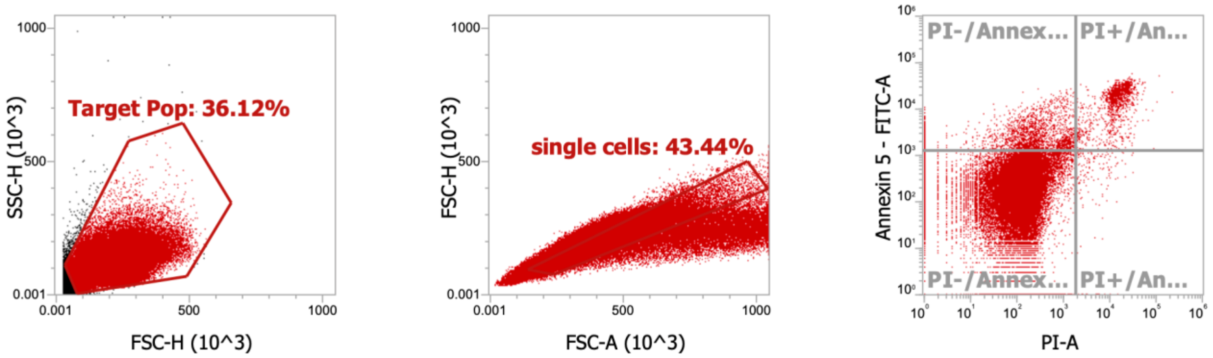

H. LPS2 - -Asn / IACS

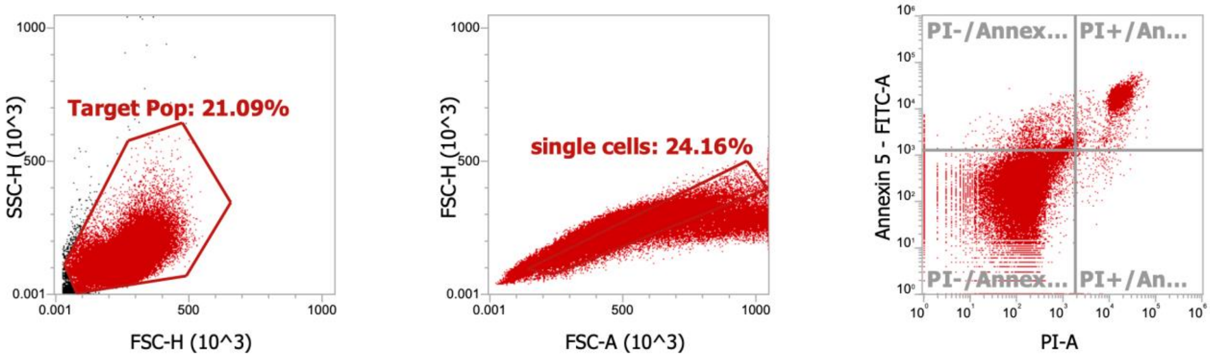

Supplement: Supplementary file 1 [file cancers-16-03031-s001.zip › cancers-3169326-supplementary.pdf]
